# Circadian Clock Proteins in Mood Regulation

**DOI:** 10.3389/fpsyt.2014.00195

**Published:** 2015-01-06

**Authors:** Timo Partonen

**Affiliations:** ^1^Department of Mental Health and Substance Abuse Services, National Institute for Health and Welfare, Helsinki, Finland

**Keywords:** affective symptoms, brown fat, cryptochrome, depressive, diurnal, genetic variation, seasonal

## Abstract

Mood regulation is known to be affected by the change of seasons. Recent research findings have suggested that mood regulation may be influenced by the function of circadian clocks. In addition, the activity of brown adipocytes has been hypothesized to contribute to mood regulation. Here, the overarching link to mood disorders might be the circadian clock protein nuclear receptor subfamily 1, group D, member 1.

There are seasonal variations in mood and behavior, including those in sleep duration, social activity, mood, appetite, weight, and energy levels that tend to reoccur year after year (1). These variations are common and present as a continuum: from individuals not having any marked variation, some having experienced the variations as a problem, and to those having the seasonal pattern of mood disorder known as seasonal affective disorder (2).

The current diagnostic criteria for seasonal affective disorder require that an episode of either recurrent major depressive disorder or bipolar disorder routinely occurs during a particular period of the year (3). Typically, in individuals with the winter type of seasonal affective disorder, or winter depression, the shortage of light during the autumn routinely induces depressive episodes, whereas the abundance of light during the spring routinely leads to remission. The treatment of choice for winter depression is bright light therapy, in which timed and repeated light exposures in the morning are administered via the eyes during the winter.

Concerning seasons and mood disorders, the clinical picture is more complex than these routine seasonal variations, as it has been noticed for centuries that a depressive episode tends to deepen and becomes life-threatening in the spring, but not to an equal extent in any other time of the year (4). There is no clear explanation to this phenomenon. A clue to understanding it may lie in reactions of the body to changes in daylight and ambient temperature during spring. Here, the circadian clocks that anticipate and try to adapt the body to the changes are in a key position. The function of brown fat being inducible in adult humans is another target to which attention is worth paying. In the following, I present my perspective on the circadian clocks and brown fat in mood disorders.

## Circadian Clocks in Mood Disorders

Nearly all people suffering from mood disorders have disruptions in circadian rhythms (5). The circadian rhythms are generated in each cell, but maintained by the master circadian clock in the neurons that are located in the suprachiasmatic nucleus of the anterior hypothalamus in the brain (6). Because the sleep–wake rhythm is dictated by the circadian clock (7), these disruptions are often seen as sleeping problems. Documentation of circadian rhythm disruptions in patients with mood disorders relies on valid markers that are generated by the master circadian clock and display a reliable circadian rhythm, such as continuous recording of core body temperature and repeated assessments of melatonin concentration (8).

Dysfunction of the proteins encoded from the circadian clock genes is hypothesized to play a role in the etiology of mood disorders (9). Here, I consider those proteins that are repressors of transcription to be most important, since they are essential to the normal function of circadian clocks (10). Among them, nuclear receptor subfamily 1, group D, member 1 (NR1D1) has a key position as a connecting node in the transcriptional and translational loops that constitute the circadian clock in a cell (11–13). Further, CRY2 and CRY1 are the key repressors in the core of the circadian clock (14–20).

Thus far, genetic association studies have suggested that variants of some, not all, circadian clock genes associate with mood disorders. Of them, *NR1D1* genetic variants have been demonstrated to associate with bipolar disorders (21–23) and depressive disorders (24, 25), *CRY2* (cryptochrome 2) with depressive disorders (26, 27) and bipolar disorders (28), and *CRY1* (cryptochrome 1) with depressive disorders (29). However, experimental studies elucidating the mechanisms of action by which the circadian clock proteins might contribute to mood disorders are missing.

## Brown Fat in Mood Disorders

A hypothesis suggests that dysfunction of the brown adipose tissue contributes to mood regulation (30). This hypothesis was based on the original finding of brown adipose tissue being clearly over-activated in two suicide cases with depressive disorder (31). On the basis of only this data, it cannot be judged whether the finding was specific or whether it is reliable.

However, it provides a basis for a view that the activation of brown adipose tissue improves cold tolerance at the cost of heat tolerance, triggering anxiety, and psychomotor agitation, and affects mood in a negative way during the spring. It provides some evidence to stimulate not only replication studies but also experimental studies to demonstrate the mechanisms of action by which the brown adipose tissue might affect mood and contribute to mood disorders.

## Role of Orphan Nuclear Receptors

Nuclear receptor subfamily 1, group D, member 1 is one of the so-called orphan nuclear receptors, while it seems to be a molecular link between the circadian clocks and mood regulation (32). Studies with Nr1d1-knockout mice agree with and support this finding, as there is up-regulation of tyrosine hydroxylase in the hippocampus (33) and increased proliferation of hippocampal neurons (34) in these mice. In these experiments, their mood-related behaviors were manifested as less anxious and less depressive.

Among the circadian clock genes, *NR1D1* is the only one that maintains its oscillation on time at the light–dark transitions as well as under constant darkness in organs throughout the body (35). Therefore, NR1D1 seems to be the principal metronome of the body. NR1D1 regulates the transcription of “the long-day gene” *TSHB* (thyroid stimulating hormone, beta), and through this action NR1D1 is also a link between the effects of light and the seasonal variation in behavior (36). Transcription of *TSHB* is induced to a greater extent about 14 h after dawn of the first long day in the spring by the increasing exposure to light (37).

Intriguingly, the circadian clock protein NR1D1 has recently been demonstrated to link the body’s circadian and thermogenic networks through the regulation of the function of brown adipose tissue (38). The physiological induction of uncoupling protein in the mitochondria by cold temperature is preceded by rapid down-regulation of *NR1D1* gene in brown adipose tissue, or in other words, the high levels of NR1D1 protein must fall before cold ambient temperature can induce uncoupling protein 1 to start producing heat and warm up the body. This switching off of the NR1D1-dependent repression is a key to the acute thermogenic response to cold and to subsequent cold tolerance.

Switching the NR1D1-dependent repression on again after it has once been switched off, however, is challenged in the spring, when the days are already long but may still be cold. Combination of long light exposure together with cold ambient temperature gives a conflicting signal of seasonal mismatch to the body (30). Having such conflict, the body is likely to continue producing heat and building up improvement in cold tolerance. If the activity of brown adipose tissue were not to be shut down as normal in the spring, it would easily become over-activated (39) and would produce excessive heat load that would give abnormal feedback from brown adipose tissue to the brain (40, 41).

## Role of Cryptochromes

*NR1D1* responds to a switch to longer days but does not immediately reset to the long-day state (42). During the resetting, the readouts of the circadian clock genes shift further away from the signal of *NR1D1*, and the magnitude of this escape is greater in Cry2-deficient than Cry1-deficient mice (43). Of the two cryptochromes, CRY2 opposes the actions of CRY1, thereby denying CRY1 from accessing to DNA targets too early (44), and in addition CRY2 opposes the actions of PER1 (45). It is the timing of peaks of *PER1* and *CRY2* expression, in particular, that varies directly with the length of the photoperiod (46, 47), and it is therefore the PER1–CRY2 and period 2 (PER2)–CRY2 protein complexes (48, 49) that control for their downstream targets during the resetting.

In addition to actions in the nucleus of a cell, the two cryptochromes act as inhibitors of adenylyl cyclase and thereby limit cyclic adenosine monophosphate production (50, 51). Interferon regulatory factor 4 is induced by cold as well as by cyclic adenosine monophosphate in adipocytes, driving up the activity of uncoupling protein 1 for heat production (52). Cryptochromes also inhibit the G protein coupled receptors activity, receptive to ligands such as vasoactive intestinal peptide and glucagon, through a direct interaction with the stimulatory G(s)alpha subunit (50). By these mechanisms, the cryptochromes might protect the individual from a depression-like state seen in conditions where dysfunction in control of the mesolimbic dopaminergic tracts leads to increased cyclic adenosine monophosphate production and increased depression-like behavior (53).

**Figure 1:**
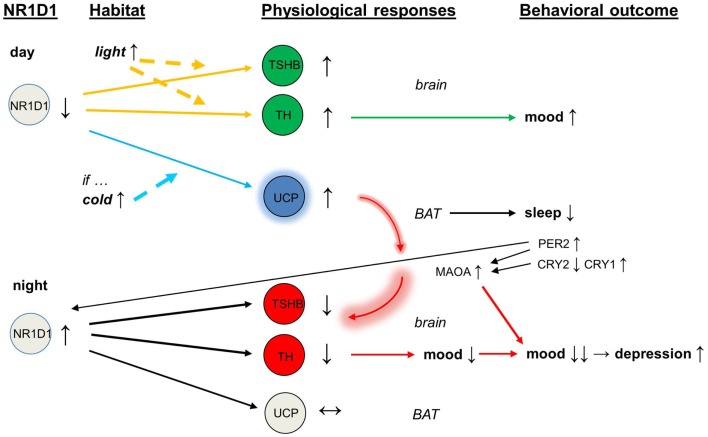
**Schematic of mood regulation affected by the circadian clock proteins (NR1D1, PER2, CRY2, CRY1) and by the activity of brown adipose tissue**. Abbreviations: NR1D1, nuclear receptor subfamily 1, group D, member 1; PER2, period 2; CRY2, cryptochrome 2; CRY1, cryptochrome 1.

With abnormal expression of CRY2, the circadian protein PER2 and the enzyme monoamine oxidase A (MAOA) would become overactive (54). The over-expression of NR1D1 inhibits the activity of tyrosine hydroxylase (32), and the over-activity of MAOA depletes dopamine release and impairs further the dopaminergic transmission. In addition, the direct interaction of PER2 with NR1D1 (55) may feedback to this vicious circle that was initiated by the overactive brown adipose tissue. In the end, mood is lowered and there is a deepening of depressive episode (see Figure 1).

## Conclusion

The loss of cryptochromes does change physiology, and dysfunction of cryptochromes may change mood. On the basis of the data presented above, *CRY2* appears to be “a mood gene.” Success in the resetting has been hypothesized to improve lowered mood in the depressed (56), whereas failure in the resetting may deepen a depressive episode any time of the year, especially in the spring. Here, the overarching link might be the circadian clock protein NR1D1.

## Conflict of Interest Statement

The author declares that the research was conducted in the absence of any commercial or financial relationships that could be construed as a potential conflict of interest.
